# Geographic isolation and environmental heterogeneity contribute to genetic differentiation in *Cephalotaxus oliveri*

**DOI:** 10.1002/ece3.9869

**Published:** 2023-03-12

**Authors:** Hanjing Liu, Zhen Wang, Yuli Zhang, Minghui Li, Ting Wang, Yingjuan Su

**Affiliations:** ^1^ School of Life Sciences Sun Yat‐sen University Guangzhou China; ^2^ College of Life Sciences South China Agricultural University Guangzhou China; ^3^ Research Institute of Sun Yat‐sen University in Shenzhen Shenzhen China

**Keywords:** *Cephalotaxus oliveri*, EST‐SSR, genetic variation, isolation‐by‐environment, outlier loci

## Abstract

Evaluating the contributions of geographic distance and environmental heterogeneity to the genetic divergence can inform the demographic history and responses to environmental change of natural populations. The isolation‐by‐distance (IBD) reveals that genetic differentiation among populations increases with geographic distance, while the isolation‐by‐environment (IBE) assumes a linear relationship between genetic variation and environmental differences among populations. Here, we sampled and genotyped 330 individuals from 18 natural populations of *Cephalotaxus oliveri* throughout the species' distribution. Twenty‐eight EST‐SSR markers were applied to analyze population genetics, for the investigation of the driving factors that shaped spatial structure. In addition, we identified the outlier loci under positive selection and tested their association with environmental factors. The results showed a moderate genetic diversity in *C. oliveri* and high genetic differentiation among populations. Population structure analyses indicated that 18 populations were clustered into two major groups. We observed that the genetic diversity of central populations decreased and the genetic differentiation increased towards the marginal populations. Additionally, the signatures of IBD and IBE were detected in *C. oliveri*, and IBE provided a better contribution to genetic differentiation. Six outlier loci under positive selection were demonstrated to be closely correlated with environmental variables, among which bio8 was associated with the greatest number of loci. Genetic evidence suggests the consistency of the central‐marginal hypothesis (CMH) for *C. oliveri*. Furthermore, our results suggest that temperature‐related variables played an important role in shaping genetic differentiation.

## Graphical abstract

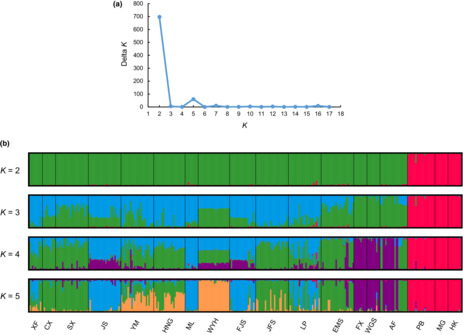

## 1 INTRODUCTION

The demographic history and population structure can be revealed by detailed characterization of the genetic variation of a species (Lee & Mitchell‐Olds, 2011). The genetic variation pattern among populations could result from the complex interaction of natural selection, genetic drift, and gene flow (Eckert et al., 2008; Hamlin & Arnold, 2015). Geographic distance and environmental heterogeneity are two key factors contributing to these processes, and further affect the distribution of allele frequencies and genetic structure in populations (Sork, 2016; Wang, 2013). Under isolation‐by‐distance (IBD), gene exchange among populations is restricted by geographic distance and barriers, resulting in the local accumulation of genetic differences that increase with geographic distance (Bradburd et al., 2013; Wright, 1943). By contrast, under isolation‐by‐environment (IBE), gene flow is limited by strong divergent selection or local adaptation to heterogeneous environment, generating positive correlations between genetic and environmental distances (Wang, 2013). These two patterns are not mutually‐exclusive and may co‐occur among populations (Manthey & Moyle, 2015). Of course, topography, geographic barriers, and postglacial recolonization may also affect the genetic structure (Cushman & Landguth, 2010; Orsini et al., 2013).

Isolation and fragmentation may influence genetic processes for plant populations within species distribution ranges (Provan & Maggs, 2012). Understanding the processes determining species range limits is important to predict the distributions of species under climate change (Afkhami et al., 2014). However, the determinants of range limits for most species are still poorly understood, mainly because such limits are caused by a complex interplay among a large number of biotic, physical, and historical factors (Roy et al., 2009). Central‐marginal hypothesis (CMH) is one of the primary evolutionary hypotheses to explain the evolution of species' range limits (Sexton et al., 2009). CMH predicts that marginal populations exhibit higher genetic differentiation and lower genetic diversity compared with the central populations (Brown, 1984). When these predictions hold, marginal populations tend to occur in reduced quality habitat resulting in the decrease of effective population size (*N*_e_), which could further reduce gene communication among populations and exacerbate genetic drift within populations. Ecologically marginal populations may be separated not only by distance from the core but also experience different biotic and abiotic environments (Munwes et al., 2010). In addition, CMH is also critical for the management and conservation of marginal populations (Hampe & Petit, 2005). Marginal populations may contain unique adaptive traits to persist under rapid climate change (Sexton et al., 2011). If marginal populations with unique adaptive potential perish in face of human‐caused climate change, the species may not be able to effectively expand back to marginal habitats or respond to range shifts. Thus, examining the CMH will confirm whether marginal populations are genetically isolated from central populations, and therefore can inform management decisions for a declining species (Micheletti & Storfer, 2015).

*Cephalotaxus oliveri* (Cephalotaxaceae) is a dioecious woody shrub or small tree, with the distinctive feature of white stomatal bands on abaxial surface of leaves. As an endemic conifer to southern China, the species has a limited distribution, only appearing in montane regions of Chongqing, southern and western Sichuan, Guizhou, Hunan, north‐western Hubei, eastern Jiangxi, northern Guangdong, and south‐eastern and north‐eastern Yunnan, China. This plant primarily grows along the sides of streams or valleys at altitudes from 300 to 1800 m and prefers warm and humid habitats (Fu et al., 1999). It contains natural anticancer alkaloid such as harringtonine and homoharringtonine, with high medicinal value (Shi et al., 2010; Xiao et al., 2019). However, its natural population size has significantly declined in recent years due to climate change and overexploitation. Ecological niche modeling of *C. oliveri* suggested that climate change will cause south‐to‐north population migration with the contraction of southern regions (Wang et al., 2016).

Several studies led to the development and successful utilization of different markers in conifer species for diversity analyses (Giles‐Perez et al., 2022; Wei et al., 2022). However, genetic variation analysis in *C. oliveri* is still in infancy. ISSRs of *C. oliveri* have been developed to investigate the genetic variation and identify the correlation between adaptive loci and variation of temperature and precipitation. However, the results of the phylogenetic tree and STRUCTURE analysis were inconsistent. Additionally, the effect of other environmental factors such as soil variables on genetic variation was not considered (Wang et al., 2016). Therefore, research based on a suitable means of detection and more environmental factors is necessary to systematically and comprehensively investigate the population structure and local adaptation of *C. oliveri*.

To explore the role of geographic isolation and environmental heterogeneity in shaping genetic variation and whether the pattern of genetic variation conforms the prediction of CMH, we used 28 EST‐SSR makers to investigate the genetic variation of 330 individuals from 18 natural populations of *C. oliveri*. The focuses of our work were to (a) assess the genetic variation and population structure of this species, and compare the genetic variation between 12 central and 6 marginal populations; (b) investigate the effects of geographic and environmental factors contributing to genetic variation; (c) identify candidate outlier loci and test for correlation between environmental variables and allelic distribution of outlier loci.

## 2 MATERIALS AND METHODS

### 2.1 Plant sampling and DNA extraction

**FIGURE 1:**
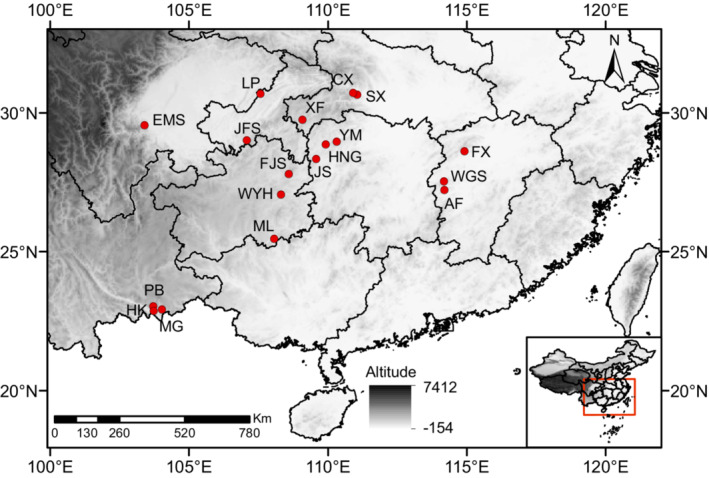
Geographic locations of 18 *Cephalotaxus oliveri* populations.

According to the geographic location, isolation, size, and habitat characteristics of *C. oliveri* populations, 18 populations were collected from the whole distribution region in southern China, including 6 marginal and 12 central populations (Figure 1; Table S1). The central populations were located in Chongqing, Sichuan, Hubei, and Hunan Provinces, because the result of ecological niche modeling showed that these areas are the highest suitable habitat for *C. oliveri* (unpublished data). While marginal populations were distributed in Yunnan and Jiangxi Provinces, and isolated from central populations, with small coverage high fragmentation and low densities. Fresh needles were collected from 330 individuals and desiccated with silica gel for subsequent use. Geographic and altitude information were obtained via GPS. Cetyltrimethylammonium bromide (CTAB) approach modified by Su et al. (2005) was operated for genomic DNA extraction. The concentration and quality of DNA were assessed using micro‐spectrophotometer (ThermoFisher) and 1.0% agarose gel electrophoresis, respectively.

### 2.2 PCR amplification and genotyping

We selected 28 polymorphic EST‐SSRs of *C. oliveri* previously developed by our laboratory (Liu, Zhang, et al., 2021), and the forward primers of these makers were labeled with two fluorescent dyes (HEX and FAM, Table S2). PCR was performed in 25 μL final solution consisting of 12.5 μL of Taq Mix Kit (Tiangen), 50 ng of genomic DNA, 0.1 μM of each primer, and appropriate ddH_2_O. Amplification procedures were carried out as follows: initial denaturation at 95°C for 5 min, then 30 cycles of 95°C for 30 s, corresponding annealing for 30 s (Table S2), and 72°C for 30 s, with a final extension step at 72°C for 10 min. The alleles with fluorescence labeling were sized using a 3730xl DNA Analyzer (Applied Biosystems: ABI). and analyzed using GeneMapper v.5 (ABI).

### 2.3 Population genetic diversity

Micro‐Checker v.2.2.3 was conducted to check the null alleles (Van Oosterhout et al., 2004). POPGENE v.1.32 was used to assess the genetic parameters of 18 populations (Yeh et al., 1999), including number of alleles (*N*_a_) and effective alleles (*N*_e_), Shannon's information index (*I*), expected (*H*_e_), and observed (*H*_o_) heterozygosity. The number of private alleles (*N*_p_) was examined using the *private_alleles* function of R package “poppr” (Kamvar et al., 2014). The inbreeding coefficient (*F*_IS_) and allelic richness (*A*_r_) were calculated using the R package “hierfstat” (Goudet, 2005). The departure from Hardy–Weinberg equilibrium (HWE) of loci in each population was tested by GENEPOP v.4.7.0 (Rousset, 2008), the Markov chain parameters were set at 10,000 dememorization steps, and the numbers of batches and iterations per batch were 20 and 5000, respectively.

### 2.4 Population genetic differentiation and structure

Analysis of molecular variance (AMOVA) was carried out in Arlequin v.3.5.1.2 to detect genetic variation (Excoffier & Lischer, 2010). The *diffCalc* function of R package “diveRsity” was run to calculate the pairwise *F*_ST_ of populations, which then was used to examine the parameter *N*_m_ according to the formula *N*_m_ = (1 − *F*_ST_) / 4*F*_ST_.

The Bayesian clustering was conducted in STRUCTURE v.2.3.4 to infer the genetic structure of 18 natural populations of *C. oliveri* (Pritchard et al., 2000). The cluster number (*K*) ranged from 1 to 18, ten runs were performed for each *K*, with Markov chain Monte Carlo (MCMC) steps of 100,000 iterations followed by a burn‐in period of 100,000 iterations. STRUCTURE HARVESTER was implemented to obtain the optimum *K* value according to the delta *K* (∆*K*) values (Earl & vonHoldt, 2012). Then, CLUMPP and DISTRUCT were conducted to plot the results (Jakobsson & Rosenberg, 2007; Rosenberg, 2004). GenAlEx v.6.5 was applied to perform principal coordinate analysis (PCoA) (Peakall & Smouse, 2012). Moreover, a UPGMA tree for 18 *C. oliveri* populations was constructed by using MEGA v. 7.0 with Nei's genetic distance (Hall, 2013).

### 2.5 Environmental variables

A total of 47 environmental variables were obtained, including 19 bioclimatic, eight ecological, and 20 soil variables. Nineteen bioclimatic variables for the current period (1950–2000, 2.5 arc‐min resolution) were extracted from WorldClim database (http://www.worldclim.org). Eight ecological variables included slope, aspect, altitude, normalized difference vegetation index (NDVI), enhanced vegetation index (EVI), percent of tree cover (PTC), leaf area Index (LAI), and fraction of absorbed photosynthetically active radiation (FAPAR). Slope and aspect were extracted from SRTM elevation data (2.5 arc‐mins resolution) and computed in ArcGIS v.10.4.1. Based on the moderate resolution imaging spectroradiometer (MODIS) dataset, the other five ecological factors were derived from the Land Process Distributed Active Archive Center (http://lpdaac.usgs.gov, 2001–2018).

The soil samples were also collected from 18 populations. The electrical conductivity and pH values of the soil samples were determined as soon as possible after water extraction from fresh soil (soil/water = 1/5 weight/volume) using a potentiometer and a conductivity meter, respectively. The moisture content of soil (fresh or air‐dried) was measured by drying at 105°C oven. The air‐dried soil samples were then smashed, and sieved through 0.2‐mm mesh for subsequent analysis. The content of soil organic matter was determined through the potassium dichromate volumetry method, which is divided by 1.724 to obtain organic carbon content. The total nitrogen (N) was evaluated by the Kjeldahl method with a mixture of K_2_SO_4_–CuSO_4_ and H_2_SO_4_ catalysts. The soil was digested in HNO_3_/HCl/HF (3:1:1) solution, inductively coupled plasma optical emission spectrometer was used to measure the total P, K, Na, Ca, Mg, Si, Al, S, Cu, Zn, Pb, Fe, and Mn. Three soil samples were collected for each population, and each sample was analyzed in triplicate. Finally, 20 soil variables of sampling sites were obtained.

To minimize the multicollinearity effects, we used the *vif.cca* function in R package “usdm” (Naimi et al., 2014) to check the variance inflation factor (VIF) of the three groups of variables, respectively, and removed the variables with VIF > 10. The final variables set contained 22 environmental variables, including six bioclimatic, seven ecological, and nine soil variables (Table S3).

### 2.6 Isolation tests

The 22 environmental variables were transformed matrix using PCA. The Euclidean distance between population pairs was calculated using the first five principal components as the environmental distance matrix. The pairwise geographic distance matrix was transformed using the geographic coordinates of populations. The pairwise genetic distance among populations using *F*_ST_ / (1 − *F*_ST_).

To explore the existence of IBD and IBE, we applied Mantel tests to assess the relationships between genetic distance and geographic or environmental distance. Then, we also used the partial Mantel test to assess the effect of one factor on genetic distance by using another factor as a covariable. The Mantel and partial Mantel tests were implemented using R package “vegan” (Dixon, 2003) based on the Pearson's correlation coefficient method with 10,000 permutations. Finally, multiple matrix regression with randomization analysis (MMRR) was performed to estimate the relative contributions of geography and environment to genetic divergence using the *MMRR* function with 999 permutations.

### 2.7 Environmental association analysis

Given that IBD and IBE were considered as useful models for describing the observed genetic differentiation (see Section 3), we performed redundancy analysis (RDA) to identify the relative importance of geography and environment on patterns of genetic variation using *rda* function of the R package “vegan.” To avoid spatial autocorrelation, distance‐based Moran's eigenvector maps (dbMEM1–dbMEM10) were applied to represent the geographic variables by transforming the coordinates of each individual using the *pcnm* function of the R package “vegan”. Hellinger transformation was used to transform EST‐SSR data into response variables using the *decostand* function in R. The 22 environmental variables and 10 geographic variables were performed forward selection separately to remove the variables that lacked explanatory power for partitioning using *ordiR2step* function of R package “vegan”. Then, RDA was performed between forward selection variables (explanatory variables) and the EST‐SSR matrix (response variables) using *rda* function. In addition, variation partitioning was also performed using the *varpar*t function in RDA. The significance of all RDA models and constrained axes was detected using the *anova.cca* function with 999 permutations.

### 2.8 Identification of outliers and association with environmental variables

We applied two methods to detect loci potentially under selection. Firstly, a Bayesian approach in BayeScan v.2.1 was performed to detect outlier loci (Foll & Gaggiotti, 2008). The parameters set for BayeScan were the default values. We calculated the q‐values to reduce the false discovery rate (FDR), therefore, the locus was recognized as an outlier when q‐value was less than .001. In addition, the hierarchical island model (HIM) implemented by Arlequin was used for coalescent simulations, and the heterozygosity and *F*_ST_ with null distributions were derived, thus estimating the locus‐specific *p*‐values. Relationships between outliers (alleles) and environmental factors were analyzed using a linear mixed‐effects model (LMM). The *lmer* function of the R package “lme4” (Bates et al., 2015) was used to construct LMM, with genetic groups as the random effect while 22 environmental variables (VIF < 10) as the fixed effect. The significance of difference was determined through a likelihood‐ratio test using the *anova.cca* function in R.

## 3 RESULTS

### 3.1 Genetic diversity

The genetic diversity indices for 18 populations of *C. oliveri* are shown in Table 1. Across the 18 populations, the *N*_a_ ranged from 1.500 (HK) to 3.714 (LP), with an average value of 2.679, and *N*_e_ varied from 1.260 (MG) to 1.922 (FJS), with an average value of 1.660. The ranges of *H*_o_ and *H*_e_ were 0.125 (HK)–0.361 (FJS) and 0.141 (MG)–0.382 (SX), respectively. SX exhibited the highest *I* (0.217), whereas the lowest was observed in MG (0.687). The *A*_r_ in each population ranged from 1.500 (HNG) to 2.909 (JFS), with a mean of 2.367. The average *F*_IS_ was 0.135, and the minimum and maximum values were −0.086 (AF) and 0.323 (XF), respectively. Except for AF, *F*_IS_ of the other populations was positive. The *N*_p_ varied from 1 (EMS, FX, and WGS) to 11 (LP and PB), and private alleles were observed in 17 populations. The genetic diversity of population MG, HK, and PB showed a lower level than that of other populations, according to the analysis of *N*_e_, *H*_o_, *H*_e_, and *I*. No evidence was found for null alleles of all loci by Micro‐Checker. Generally, except for Co164, all other loci significantly deviated from HWE (Table S4).

**TABLE 1:** Genetic diversity for 18 populations of *Cephalotaxus oliveri*.

Population	*N*_a_	*N*_e_	*I*	*H*_o_	*H*_e_	*N*p	*A*_r_	*F*_IS_
*Central*								
XF	2.321	1.568	0.474	0.264	0.284	3	2.309	0.323
CX	2.464	1.745	0.557	0.304	0.337	3	2.464	0.116
SX	3.500	1.846	0.687	0.311	0.382	2	2.224	0.176
JS	3.250	1.884	0.680	0.330	0.373	2	2.803	0.019
YM	3.357	1.747	0.632	0.307	0.350	4	2.000	0.164
HNG	3.036	1.747	0.569	0.284	0.314	6	1.500	0.173
ML	2.357	1.798	0.563	0.304	0.348	3	2.495	0.087
WYH	3.000	1.626	0.518	0.284	0.281	3	2.284	0.200
FJS	3.321	1.922	0.676	0.361	0.375	5	2.782	0.146
JFS	2.571	1.742	0.558	0.279	0.333	3	2.909	0.126
LP	3.714	1.771	0.653	0.317	0.341	11	1.536	0.038
EMS	2.607	1.654	0.484	0.239	0.274	1	2.357	0.148
Mean	2.958	1.754	0.587	0.299	0.333	3.833	2.305	0.143
*Marginal*								
FX	2.000	1.456	0.393	0.196	0.251	1	2.317	0.311
WGS	2.393	1.584	0.503	0.339	0.311	1	2.807	0.216
AF	2.607	1.628	0.509	0.179	0.291	5	2.393	−0.086
PB	2.679	1.556	0.499	0.216	0.287	11	2.440	0.077
MG	1.536	1.260	0.217	0.132	0.141	2	2.321	0.099
HK	1.500	1.339	0.237	0.125	0.153	0	2.669	0.107
Mean	2.119	1.470	0.393	0.198	0.239	3.333	2.491	0.120
Overall mean	2.679	1.660	0.523	0.265	0.301	3.667	2.367	0.135

Abbreviations: *A*_r_, allelic richness; *F*_IS_, inbreeding coefficient; *H*_e_, expected heterozygosity; *H*_o_, observed heterozygosity; *I*, Shannon's information index; *N*_a_, number of alleles; *N*_e_, number of effective alleles; *N*_p_, number of private alleles.

### 3.2 Genetic variation and population structure

**FIGURE 2:**
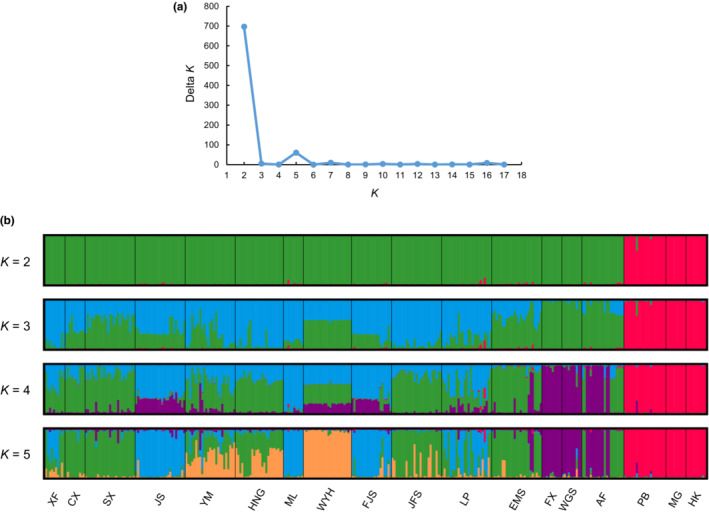
The genetic structure of the 18 *Cephalotaxus oliveri* populations in southern China. (a) Estimation of population structure using delta *K* (∆*K*) with the number of clusters (*K*) ranging from 1 to 18. (b) Estimation of population structure of *C. oliveri* using the STRUCTURE analysis.

**FIGURE 3:**
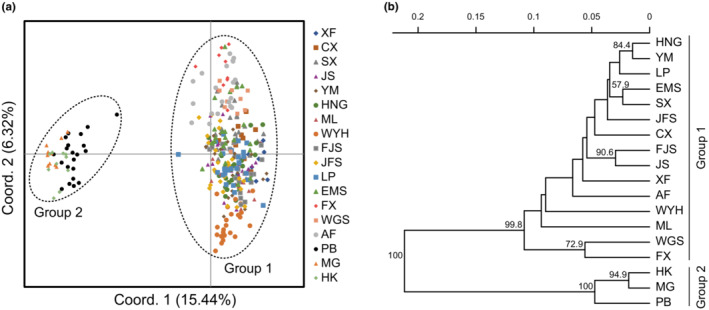
Genetic variation and relationships among the *Cephalotaxus oliveri* populations. (a) Principal coordinate analysis (PCoA) based on pairwise genetic distance. (b) UPGMA dendrogram based on Nei's genetic distance.

The STRUCTURE results showed that the ∆*K* was the highest peak when *K* = 2, therefore, the optimum number of genetic clusters for populations was suggested as 2 (Figure 2a). The 18 populations of *C. oliveri* included two main genetic groups when *K* = 2. Group 1 included 15 populations (XF, CX, SX, JS, YM, HNG, ML, WYH, FJS, JFS, LP, EMS, FX, WGS, and AF), and group 2 included 3 populations of Yunnan Province (PB, MG, and HK) (Figure 2b). The results of PCoA showed that 330 individuals of 18 populations could also be clustered into two main groups, which was in accordance with the STRUCTURE analysis. The first two PCoA axes accounted for 15.44% and 6.32% of the total variation, respectively (Figure 3a). Moreover, the results of the UPGMA clustering analysis further supported the STRUCTURE analysis and PCoA results (Figure 3b).

The AMOVA results for all populations showed that 26% and 74% of the total genetic variation was among and within populations, respectively (*p* < .01). The *F*_ST_ was 0.260 (*F*_ST_ > 0.25; *p* < .0001), suggesting that there was significant genetic differentiation among populations (Table 2). Furthermore, AMOVA results of the two genetic groups clustered by the STRUCTURE results exhibited that 30% of the total variation was explained by the differentiation between group 1 and group 2 (*F*_ST_ = 0.433, *p* < .01), while differences among populations within two groups (*F*_SC_ = 0.185, *p* < .01) and within populations (*F*_CT_ = 0.304, *p* < .01) accounted for 13% and 57% of the variation, respectively, indicating low genetic differentiation among populations within these two groups.

**TABLE 2:** Analysis of molecular variance (AMOVA) of *Cephalotaxus oliveri* populations.

Source of variation	df	Sum of squares	Variance components	Percentage variation
Among populations	17	1029.301	1.54393	25.99448
Within populations	642	2821.919	4.39551	74.00552
Total	659	3851.22	5.93944	
Fixation Index	*F*_ST_ = 0.25994			

The pairwise *F*_ST_ values were 0.035–0.606, and these values for most population pairs were high (>0.15), suggesting high genetic differentiation. The lowest values were observed between MG and FX, MG and XF, HK and FX, and HK and XF, while the greatest was observed between populations YM and HNG, YM and LP, and CX and SX. In addition, the mean value of pairwise *F*_ST_ among populations from group 1 was 0.197, and that of group 2 was 0.180, both were lower than the value (0.475) between group 1 and group 2 (Table S5). Accordingly, the values of gene flow (*N*_m_) within each group were higher than that across the groups (1.326 [group 1] and 1.206 [group 2] vs. 0.286 [between group], Table S5).

### 3.3 Genetic comparison of central and marginal populations

We also evaluated the intrapopulation genetic diversity and differentiation between central and marginal groups. The average *A*_r_ value of populations in the marginal group (2.491) was higher than populations in the central group (2.305). The *H*_o_ and *H*_e_ in the marginal populations (0.198 and 0.239) were lower than the central populations (0.299 and 0.333). Overall, except for *N*_p_ and *A*_r_, other indices (*N*_a_, *N*_e_, *I*, *H*_o_, and *H*_e_) for the central group were significantly greater than that for the marginal group (*p* < .05; Table 1). Moreover, the pairwise *F*_ST_ for populations of the central group (mean *F*_ST_ = 0.162) was significantly lower than those for populations of the marginal group (mean *F*_ST_ = 0.395, *p* < .05; Table S5). The gene flow for central populations (mean *N*_m_ = 1.641) was higher than marginal populations (mean *N*_m_ = 0.555).

### 3.4 IBD and IBE

**FIGURE 4:**
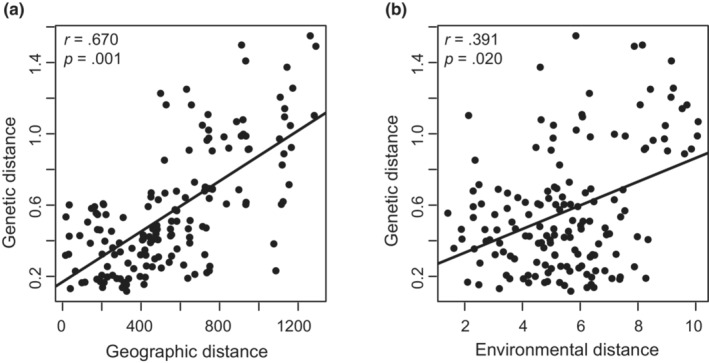
The relationship between genetic and geographic or environmental distances based on Mantel tests. (a) The test of isolation‐by‐distance (IBD). (b) The test of isolation‐by‐environment (IBE).

The Mantel test detected significant relationships between genetic distance and geographic (*r* = .670, *p* = .001) or environmental (*r* = .391, *p* = .020) distance, indicating significant patterns of IBD and IBE among populations of *C. oliveri* (Figure 4; Table S6). The partial Mantel tests showed that when controlling the influence of another factor, the relationship between genetic variation and geographic distance remained significant (*r* = .609, *p* = .001), whereas the association between genetic distance and environmental distance was nonsignificant (*r* = .183, *p* = .142; Table S6). In addition, MMRR with the separate effect of geographic or environmental factor on the genetic distance both showed significant correlations, and the contribution of environmental factor had a relatively higher rate than that of the geographic distance (*β*_Geo_ = .0007, *p* = .001; *β*_Env_ = .0743, *p* = .017; Table S6). Whereas considering geography and environment simultaneously, the result suggested that genetic differentiation of *C. oliveri* was significantly related to geographic distance (*β*_Geo_ = .0007, *p* = .001), while the effect of environmental distance on genetic differentiation was nonsignificant (*β*_Env_ = .0276, *p* = .215).

### 3.5 RDA

**FIGURE 5:**
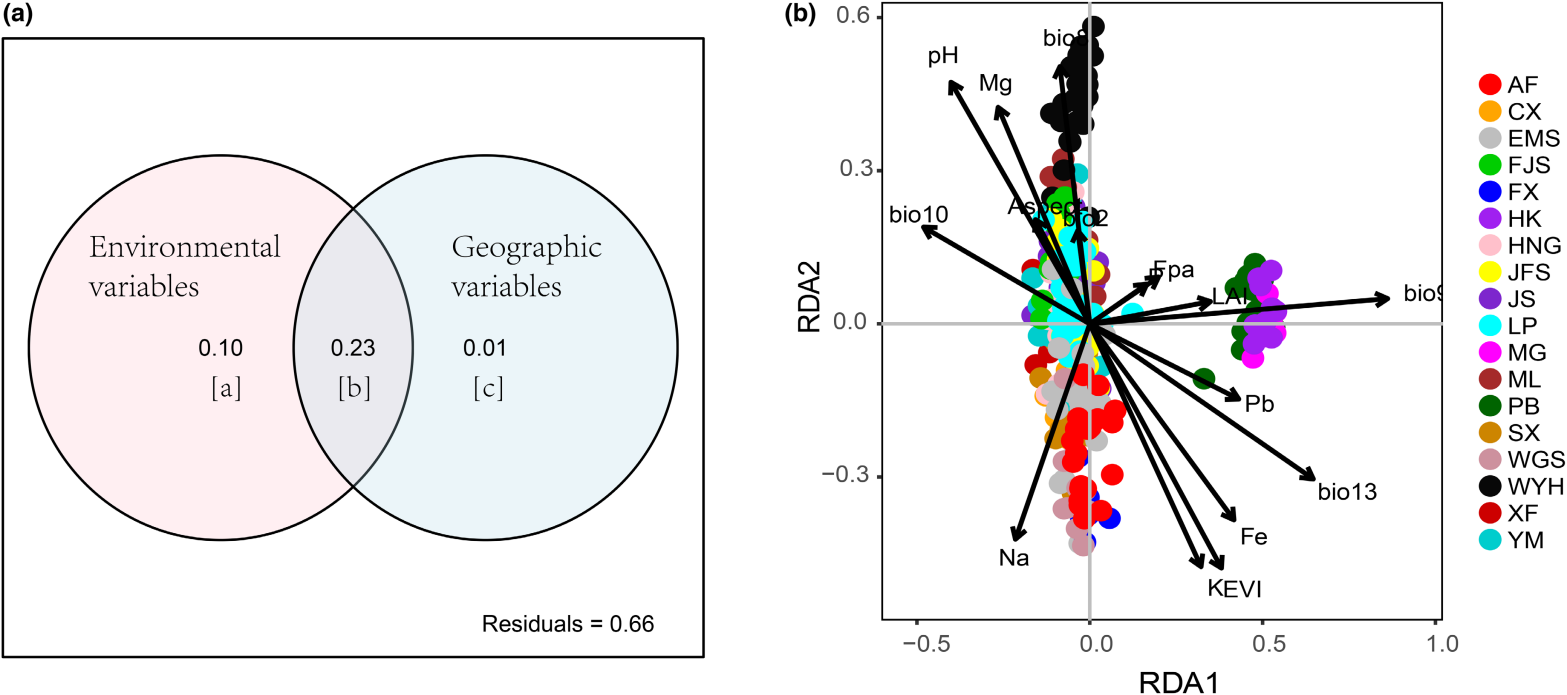
(a) The result of redundancy analysis (RDA) on RDA axis 1 and axis 2. (b) Plot of variation partitioning results.

Due to the strong autocorrelation between environmental difference and geographic distance (*r* = .399, *p* = .001; Table S6), the individual effects of geography and environment and their combined effect on genetic differentiation were further analyzed by RDA. Forward selection revealed that 9 geographic variables and 16 environmental variables were significant predictors of genetic variation (Table S7). The results of variation partitioning demonstrated that geographic and environmental variables explained a large proportion of genetic variation. Geography and environment jointly explained 33.90% of variation ([a + b + c]), while 0.60% and 9.88% of the variation were separately explained by geography ([c]) and environment ([a]), respectively (*p* < .05; Table 3; Figure 5a), suggesting that the environmental factors were more important. Among the 16 environmental variables, soil Fe, Pb, P and K content, and bio8 were the top five environmental variables that explained genetic variation. Seventeen axes (RDA1 to RDA17) explained 33.90% of the total variance. RDA1 and RDA2 were mainly correlated with bio9 and bio13, and bio8 and EVI, respectively (Figure 5b).

**TABLE 3:** Redundancy analyses (RDAs) of *Cephalotaxus oliveri*.

	Adjusted *R* ^2^	*F*	*p*
[a + b] Gen. ~ Env.	.3329	11.263	.001
[c + b] Gen. ~ Geo.	.2402	12.556	.001
Individual fractions			
[a]	.0988	6.9761	.001
[c]	.006	3.8458	.001
[b]	.2342		
[a + b + c]	.339	10.923	.001
Total unexplained	.661		

Abbreviations: Env, environmental variables (16 environmental variables); Gen, EST‐SSR data matrix; Geo, geographic variables (9 dbMEM variables).

### 3.6 Identification of putatively adaptive loci

**FIGURE 6:**
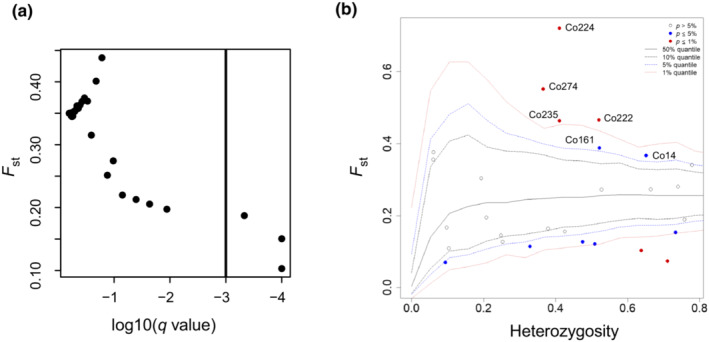
(a) Outlier loci detected by BayeScan. (b) Outlier loci detected by the hierarchical island model implemented in Arlequin.

Three loci were identified as balance selection loci by Bayesian analysis in BayeScan, while no positive selection loci were detected (Figure 6a). By contrast, 13 outlier loci with significant *p*‐value were observed in Arlequin (Figure 6b), of which potential positive selection signatures were detected in six loci: Co161, Co235, Co274, Co14, Co224, and Co222, which were annotated as protein tyrosine kinase, phosphatidylinositol 4‐kinase gamma 6, transcription factor RF2b, DEAD/DEAH box helicase, B‐box zinc finger protein 22, and chloroplast envelope membrane protein, respectively.

A total of 78 associations were identified between 31 alleles (six loci) and 19 environmental variables, 27 of which were related to bioclimatic variables, 16 were related to ecological variables, and 35 to soil variables (Table S8). The main predictors associated with loci were bio8 (8 loci), altitude (7), pH (7), Mn (7), bio12 (annual precipitation, 6), and bio13 (6). However, bio10 (mean temperature of warmest quarter), EVI, and slope were not significantly associated with all loci. Both Co14_267 and Co222_217 were significantly associated with six variables (bio8, bio9, bio12, bio13, soil pH, and K content).

## 4 DISCUSSION

### 4.1 Genetic variation and population structure

The level and distribution of genetic diversity of natural species largely represent their evolutionary potential and adaptive ability to future environmental changes (Gao et al., 2012; Jump & Penuelas, 2005). *H*_e_ is a primary index to measure gene diversity and has been used to measure the level in several studies (Slatkin & Barton, 1989; Tong et al., 2020). In this study, the average *H*_e_ was 0.301 at the species level, indicating moderate genetic diversity for *C. oliveri*. Compared with the results of other endemic species in east Asia, the genetic diversity for *C. oliveri* was higher than *Amentotaxus formosana* (*H*_e_ = 0.199; Li et al., 2016) and *P. koraiensis* (*H*_e_ = 0.23; Wei et al., 2022); and close to *Pinus squamaia* (*H*_e_ = 0.356; Mao et al., 2015) and *A. yunnanensis* (*H*_e_ = 0.334; Li et al., 2016); while lower than that of *Quercus chungii* (*H*_e_ = 0.541; Jiang et al., 2019), *Populus adenopoda* (*H*_e_ = 0.418; Fan et al., 2018) and *P. massoniana* (*H*_e_ = 0.5483; Mei et al., 2021). Genetic diversity of species may be relative to its characteristics and external factors, such as breeding system, genetic drift, and selection (Zhou et al., 2020). As a conifer species with scattered distribution, *C. oliveri* primarily grew in montane regions with humid and shady environments. Many isolated populations in fragmented habitats were faced with inbreeding and genetic drift, which was also supported by our results of *F*_IS_ and HWE analysis. Meanwhile, the long generation time and evolutionary process with a low mutation rate might be responsible for the moderate level of diversity of *C. oliveri* (Hewitt, 2000). In addition, *C. oliveri* underwent repetitious climatic changes especially Quaternary glaciations, which would result in loss of distributed range and thus loss of crucial genetic diversity (Hewitt, 2000). Finally, human activities might also lead to decrease in population size.

Based on Wright's (1965) opinion, the genetic differentiation level among populations is high when the *F*_ST_ > 0.25. The genetic differentiation for *C. oliveri* was high (0.260), which illustrated that a large proportion of the genetic variation occurred within populations. Our results were consistent with the finding that the genetic variation of perennial cross‐pollinated plants mainly existed within the population (Wu et al., 2021; Zhou et al., 2020). In addition, the genetic differentiation of *C. oliveri* in this study was lower than that determined by ISSR (0.396) and chloroplast sequences (0.639), mainly because of the limited sampling size of populations and differences in molecular markers. The *N*_m_ of *C. oliveri* populations was 1.018 (Table S5). As a wind‐pollination species, *C. oliveri* grows in understory and wind only can disperse its pollen over a short distance, making it difficult to genetically communicate with other populations over long distances (Zhang et al., 2005). Moreover, due to the seed gravity and external factors, most seeds are naturally scattered near the mother plant, which also limits the diffusion distance of *C. oliveri* seeds. Squirrels and birds can disperse a few seeds, but the dispersal distance is relatively limited (Cain et al., 2000; Lu, 2006).

Results from STRUCTURE, PCoA, and UPGMA indicated two distinct genetic clusters of *C. oliveri*. The three populations of group 2 were geographically closest and were on the same distribution continuum, while the distance from group 1 was more than 500 km. The results of IBD showed that the geographic distances between populations had a significant contribution to the genetic divergence of *C. oliveri*, indicating the fundamental role of spatial distance in the genetic divergence of *C. oliveri* populations. Wumeng Mountains (WM), about 4200 meters high in elevation, have been illustrated to play a major role in the population divergence of the species (Gong et al., 2011; Zhang et al., 2011). Group 1 and group 2 in this study were divided into different groups on the south or north side of WM, the pair *F*_ST_ values between two sides of WM were higher than between populations on each side of WM. On the contrary, the gene flow between two sides of WM was lower than between populations on each side of WM. Therefore, the patterns of *F*_ST_ and gene flow suggested that the WM might act as a physical barrier, interrupting the genetic communication between populations on both sides of the WM, which was also supported by the previous research result of chloroplast DNA (Wang et al., 2014). In addition to geographic distance and barriers, environmental differences may also be the critical factor affecting effective gene exchange. In this study, we also detected the IBE pattern of *C. oliveri*, indicating the important influence of the environmental difference in the survival and reproduction of individuals. The same signature of the IBE pattern was also found in *P. cembra* (Toth et al., 2019), *P. taeda* (Lu et al., 2019), and *Pseudotaxus chienii* (Liu, Wang, et al., 2021). These results imply that geographic distance and environmental heterogeneity are major factors resulting in the genetic divergence in *C. oliveri*.

### 4.2 CMH

The genetic diversity of many species was observed to decline towards the margins of the species range (Lesica & Allendorf, 1995). Previous review reported that 64.2% of studies (86 of 134) showed the expected decrease in genetic diversity, and 70.2% detected increased differentiation towards range margin (Eckert et al., 2008). Our results found convincing evidence to support the CMH in *C. oliveri* from southern China. In the sampled populations covering its distribution range, we detected a decline in genetic diversity for all genetic indices except two, while the genetic differentiation from the center towards margin range showed an increasing trend. The reasons may include isolation, limited gene flow, increased fragmentation, and genetic drift (Pfeifer et al., 2009). Additionally, a random decrease in the genetic diversity of marginal populations may restrict the evolutionary potential of species, and inhibit the ability of the species adapting to environmental conditions outside the range limits (Blows & Hoffmann, 2005). Similar patterns consistent with the CMH have been detected in *Euptelea pleiospermum* (Wei et al., 2016), *Abies sachalinensis* (Kitamura et al., 2020), and *Taxus wallichiana* (Liu, Wang, et al., 2019), but some species in subtropical China have not found such evidence, such as *P. massoniana* (Ge et al., 2012), *Liriodendron chinense* (Yang et al., 2016), and *Castanopsis eyrei* (Shi et al., 2014). This suggests that different plant species in the same area do not always in accord with genetic patterns of CMH (Wei et al., 2016).

The species distribution model indicated that the marginal habitats for *C. oliveri* were located in south‐western and south‐eastern China, while the suitable habitat was at the junction of Hunan and Hubei Provinces (unpublish data), which were predicted as two potential refugia for *C. oliveri* (Wang et al., 2014). Due to low genetic diversity and relative genetic isolation, marginal populations will not expand beyond their current borders, despite the existence of suitable habitats. It is predicted that the distribution range of *C. oliveri* will shift radically or shrink under global warming, when the majority of marginal populations have low genetic diversity and effective population sizes. Thus, population genetics in combination with ecological niche modeling indicated that marginal populations, especially south‐western populations are important for conservation. Our results may potentially influence management decisions for *C. oliveri* in face of climate change.

### 4.3 Identification of environmental predictors and putatively adaptive loci

Detection of IBD and IBE in *C. oliveri* by the Mantel test revealed that geography and environment were important for the observed genetic variation. RDA was further implemented to distinguish the separate roles of geographic and environmental factors. The result of RDA suggested that the influence of the environment was more obvious than geography in the genetic differentiation of *C. oliveri*. The IBE pattern was also detected in *P. chienii* (Liu, Wang, et al., 2021) and *P. tabuliformis* (Xia et al., 2018). Sexton et al. (2014) found that a total of 74% of 70 studies showed the signature of IBE patterns, consisting of 37% only exhibited a significant IBE model but no evidence for IBD. The IBE pattern may arise from natural selection among populations during the local adaptation of these populations to heterogeneous habitat environments (Nosil et al., 2005; Wang & Bradburd, 2014). In this study, differential adaptability to diverse habitats offers a preferable explanation compared with IBD in *C. oliveri*.

With the 16 environmental variables retained, the soil Fe, Pb, P and K content, and bio8 were found to be important determinants of genetic differentiation through the forward selection of RDA. Six outliers were identified to be strongly related to environmental variables by LMM. Bio8, altitude, soil pH, soil Mn content, bio12, and bio13 were associated with the most loci. Both the RDA and LMM method showed strong divergent selection signals associated with the temperature‐related variable. Temperature is a key factor affecting the phenology and physiology of species, their distribution and range, and structure and dynamics of ecosystems (Nyakatya & McGeoch, 2008). Temperature has been verified to be an important selective driver of adaptive variation in different conifers (Di Pierro et al., 2016; Jia et al., 2020). Moreover, the successful growth and reproduction of plants require various mineral nutrients, which must be absorbed from the soil matrix (DalCorso et al., 2014). Soil Fe, Pb, P, and K content were also important variables to influence the genetic variation of *C. oliveri*. P and K are essential nutrients for plant growth. Fe is involved in many key cellular metabolic processes, including respiration, photosynthesis, chlorophyll biosynthesis, and synthesis of heme iron and iron–sulfur clusters. However, Pb is a nonessential element and harmful to plants.

In this study, we identified six loci under positive selection and two of which encode TFs. Locus Co224 was significantly associated with soil P, Mg, and Mn content, and its encoded sequence is similar to B‐box‐type zinc finger protein (BXX proteins 22). Zinc finger proteins are a large family of transcription factors in plants and play a key role in regulating growth, development, and response to abiotic stresses in plants (Khanna et al., 2009). BBX proteins belong to a subfamily of zinc finger proteins that include one or more B‐Box domains and are considered to participate in protein–protein interactions (Gangappa & Botto, 2014). BBX genes have been isolated and identified from plants such as *Betula luminifera* (Dou et al., 2015), *Arabidopsis thaliana* (Khanna et al., 2009), rice (Huang et al., 2012), and *Malus domestica* (Liu, Dai, et al., 2019). Most of these TFs are related to plant growth, development, and stress resistance. Locus Co274 is located in a region tightly linked to the transcription factor RF2b that has high amino acid sequence similarity with RF2a, and both belong to the bZIP TFs family. The bZIP TFs regulate processes such as light and stress signaling, flower development, and seed maturation (Lindemose et al., 2013; Yang et al., 2019). RF2a and RF2b were confirmed to be predominantly localized to vascular tissues. However, their subcellular localization and the accumulation in different organs were different (Dai et al., 2004). The wheat RF2 subfamily bZIP protein gene TabZIP3 was induced by salt or drought treatment and its overexpression caused improved salt stress tolerance (Guo et al., 2019). Moreover, Co14 is located in one gene of DEAD/DEAH box helicase family, which plays an important role in plant growth and development, disease resistance, and stress tolerance (Li et al., 2008; Nawaz et al., 2018). Several researches confirmed that DEAD‐box family was involved in abiotic stress response in plants including rice (Tuteja et al., 2013) and tomato (Zhu et al., 2015). Therefore, these loci may participate in the responses of *C. oliveri* to different abiotic stresses and play a major regulatory role in the response to stress.

## 5 CONCLUSIONS

This study used EST‐SSR markers to investigate the population genetics of *C. oliveri*, an endemic conifer species in subtropical China. We found that the existing *C. oliveri* populations maintain a moderate level of genetic diversity and a high level of genetic differentiation. Population structure analysis identified two genetic groups. The results also supported the CMH that marginal populations exhibited lower genetic diversity and higher genetic differentiation than central populations in *C. oliveri*. Furthermore, we detected significant IBD and IBE patterns in this study, and environmental differences had more contributions in shaping population differentiation than geographic distance. We identified six outlier loci that were related to environmental variables, bio8 was associated with the most loci. Our study gained abundant genetic resources of *C. oliveri* and provided insights into local adaptation.

## AUTHOR CONTRIBUTIONS

**Hanjing Liu:** Formal analysis (lead); methodology (lead); software (lead); validation (lead); visualization (lead); writing – original draft (lead). **Zhen Wang:** Data curation (equal); formal analysis (equal); software (equal); visualization (equal). **Yuli Zhang:** Data curation (equal); formal analysis (equal); methodology (equal); validation (equal). **Minghui Li:** Formal analysis (equal); software (equal); validation (equal); visualization (equal). **Ting Wang:** Data curation (equal); funding acquisition (equal); methodology (equal); supervision (equal); writing – review and editing (equal). **Yingjuan Su:** Data curation (equal); funding acquisition (lead); methodology (equal); project administration (lead); supervision (lead); writing – review and editing (lead).

## FUNDING INFORMATION

This work was supported by the National Natural Science Foundation of China (31872670 and 32071781), Guangdong Basic and Applied Basic Research Foundation (2021A1515010911), Science and Technology Projects in Guangzhou (202206010107), and Project of Department of Science and Technology of Shenzhen City, Guangdong, China (JCYJ20190813172001780 and JCYJ20210324141000001).

## CONFLICT OF INTEREST STATEMENT

The authors declare that there are no conflicts of interest.

## Supporting information


Appendix S1

## Data Availability

Individual genotype data of *C. oliveri* are available on Dryad (https://doi.org/10.5061/dryad.qbzkh18mp).
