# Supplementary material for: Geographic isolation and environmental heterogeneity contribute to genetic differentiation in Cephalotaxus oliveri
Source: Ecol Evol. 2023 Mar 12;13(3):e9869. doi: 10.1002/ece3.9869 (PMC10008294; doi:10.1002/ece3.9869)
Supplement: Supplementary file 1 — Appendix S1 [file ECE3-13-e9869-s001.docx]

**Supporting Information**


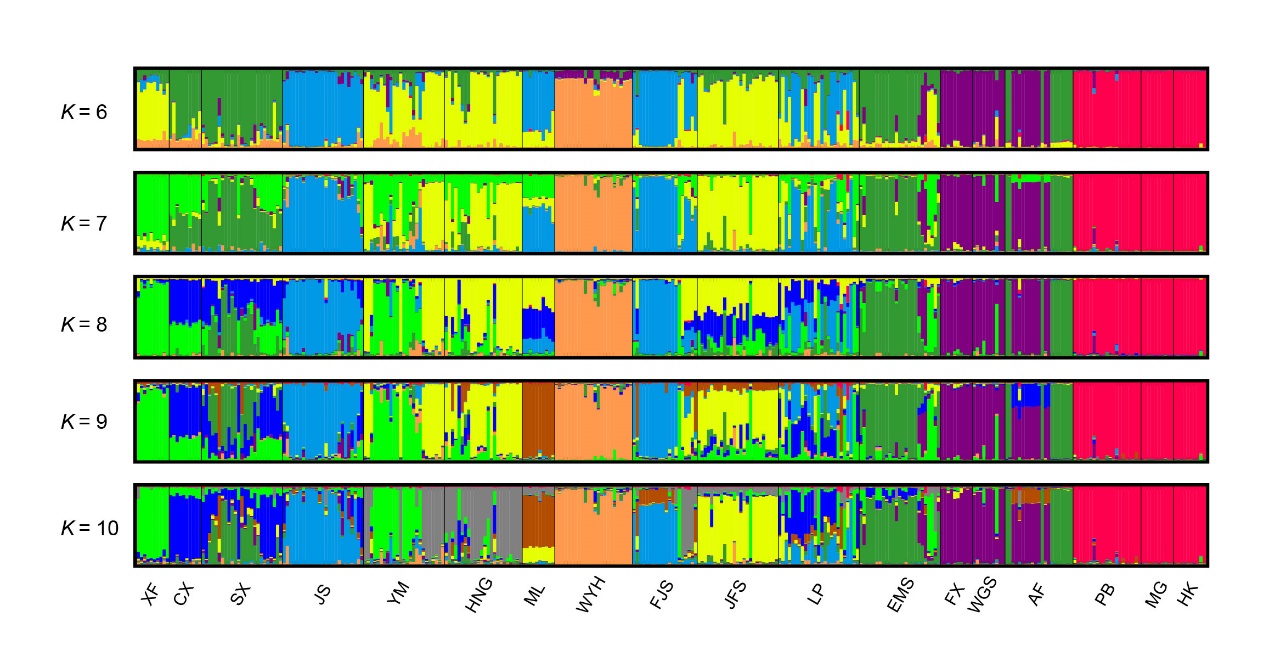


Figure S1 Genetic clustering under *K* = 6 -10 based on STRUCTURE results

Table S1 The geographical information of 18 *Cephalotaxus oliveri* populations

| Location | ID | Sample size | Latitude(N) | Longtitude(E) | Average | Sampling Date |
| --- | --- | --- | --- | --- | --- | --- |
| Xianfeng, Hubei | XF | 10 | 29°45′06.35″ | 109°4′35.43″ | 697 | 2019.6 |
| Chexi, Hubei | CX | 10 | 30°39′53.01″ | 111°3′22.93″ | 231 | 2019.6 |
| Sixi, Hubei | SX | 25 | 30°43′36.37″ | 110°54′43.53″ | 340 | 2019.6 |
| Jishou, Hunan | JS | 25 | 28°21′00.55″ | 109°35′06.03″ | 291 | 2019.6 |
| Yongmao, Hunan | YM | 25 | 28°58′13.20″ | 110°18′33.49″ | 533 | 2019.6 |
| Hanigong, Hunan | HNG | 24 | 28°51′49.97″ | 109°55′06.50″ | 347 | 2019.6 |
| Maolan, Guizhou | ML | 10 | 25°28′11.64″ | 108°4′04.77″ | 635 | 2019.6 |
| Wuyanghe, Guizhou | WYH | 24 | 27°3′24.64″ | 108°18′38.04″ | 508 | 2019.6 |
| Fanjingshan, Guizhou | FJS | 20 | 27°48′25.24″ | 108°35′44.51″ | 739 | 2019.6 |
| Jingfoshan, Chongqing | JFS | 25 | 29°1′23.21″ | 107°5′05.20″ | 851 | 2019.6 |
| Liangping, Chongqing | LP | 25 | 30°41′50.10″ | 107°34′17.58″ | 549 | 2019.6 |
| Emeishan,Sichuan | EMS | 25 | 29°33′12.39″ | 103°23′45.46″ | 976 | 2019.6 |
| Fengxin, Jiangxi | FX | 10 | 28°37′44.50″ | 114°54′47.70″ | 741 | 2019.7 |
| Wugongshan, Jiangxi | WGS | 10 | 27°32′11.90″ | 114°10′31.10″ | 625 | 2019.7 |
| Anfu, Jiangxi | AF | 21 | 27°13′48.50″ | 114°11′24.30″ | 468 | 2019.7 |
| Pingbian, Yunnan | PB | 21 | 23°1′36.85″ | 103°42′22.16″ | 670 | 2019.7 |
| Maguang, Yunnan | MG | 10 | 22°54′56.46″ | 104°1′00.60″ | 1682 | 2019.7 |
| Hekou, Yunnan | HK | 10 | 22°52′17.13″ | 103°42′59.62″ | 1435 | 2019.7 |

Table S2 The information of 28 pairs of EST-SSR primers

| Locus | Forward primers (5’-3’) | Reverse primers (5’-3’) | Repeat motif | T(℃) | Fluorescent dye | SSR position |
| --- | --- | --- | --- | --- | --- | --- |
| Co258 | AGGAGCCGGAACAGGAAATG | AAACACAATGTGCTCCACGC | (TGGAAA)5 | 61 | FAM | utr5 |
| Co229 | TGCTTGGCGGGTATGATGTT | AGGTCAGGATTCGGGAGGAA | (TGATTG)6 | 61 | HEX | cds |
| Co161 | CGACGCCCATGAGACTCAAT | CAGCCTCTCCTACAACTGGC | (TGGCAG)5 | 61 | FAM | utr5 |
| Co235 | TGCATGCAGGCTAAGTTGGA | TGCTTCCACAGAGTCGTGTC | (AGG)6 | 61 | HEX | cds |
| Co268 | TCTTCAACAACCGCGGAAGA | CAAGGAAATCCAGGAGGCGT | (CGC)6 | 61 | FAM | utr5 |
| Co264 | AAGCCCAAGCGGAGTTAGAG | TGCATCTGTTCCACCAGGTC | (AG)10 | 61 | HEX | utr5 |
| Co266 | CTGGGTGGGATTGCTCCAAT | TGGACGCAAGAAGCCTTCAT | (GGAAGA)5 | 61 | FAM | cds |
| Co111 | AGCCCAGAAGATTTGCATGGA | TGAGGTCTGCGTTTGAGGAC | (AAAC)5 | 61 | HEX | utr3 |
| Co146 | GATCGGCAATGCTAATGGCG | TGTTTCTCTCTGCACCGGTC | (TGCCAA)6 | 61 | HEX | cds |
| Co267 | GGACGACAGCAATGGCATTC | GGCGAATGTGTTGCTGGTTT | (CAG)8 | 61 | HEX | utr5 |
| Co271 | AAGTTGCAGAAGAGGAGGGG | CCTCCTCTGCTTCCTCCTCT | (GAGGAA)5 | 61 | FAM | cds |
| Co274 | TGGCCTGATACGATTGTGCT | TGTGTGAAAAGGAGCCCGAA | (TTAT)5 | 61 | HEX | utr5 |
| Co75 | GTGGAACCTCAGTCTGCGAA | CACTCATCTTCCGCCTCCTC | (AGG)7 | 61 | FAM | cds |
| Co20 | TACTGGTCCTCCTAGGCCAC | GCCCATCTGAGTTCCAGGAG | (CCA)6 | 61 | HEX | cds |
| Co14 | TGTTGGGGCGGAATAAGCAT | AGAGCACTGGTTGATGGCAA | (CCGTTG)6 | 61 | FAM | utr5 |
| Co82 | ACAAGTGTACGTGTGGCCAA | TCTAACGCAGGACAGATCGC | (TCT)6 | 61 | HEX | cds |
| Co43 | TAACCTAAGAGGGAGGGGGC | GGGCGTCTGGGATCCATTAG | (GAC)8 | 61 | FAM | utr5 |
| Co22 | AGCCCAGAAGATTTGCATGGA | TGAGGTCTGCGTTTGAGGAC | (AAAC)5 | 61 | HEX | utr3 |
| Co228 | TGGTGTGGTGTGGTGTACTG | CGACACCACAACGCCTTTTT | (GATG)5 | 61 | FAM | utr5 |
| Co257 | GATCAATGCTCCCCTGCTGT | TCTCCACTACGGCACTCTCT | (CAC)8 | 61 | HEX | utr5 |
| Co66 | TTTCCCCACCTCTTCCCAGA | CACTGCTCTCACTGCCTTCA | (AGC)6 | 61 | FAM | cds |
| Co224 | GCCTTCAACCCGTCAACAAC | ACCATAGTCCGGGTGAGGAA | (AGGAGC)5 | 61 | HEX | cds |
| Co77 | GCCACTGTTATTCTGCAGCG | AGCGATGGTAACGAGCCTTC | (GCC)7 | 61 | FAM | utr5 |
| Co236 | CCAGAGACCCCAGCAAGAAG | CGAAGGAGGTTTTGGAGGCT | (AGA)8 | 61 | HEX | cds |
| Co222 | GCAGCGCCATTATCAAGTGG | GGGTATCTGCCTCGTCACAC | (TGGTAC)5 | 61 | FAM | utr5 |
| Co244 | GCTGAAATGGGGGACTCCAA | GATCTTTGCGCCCTGTTTCC | (GAA)6 | 61 | HEX | cds |
| Co234 | TGCAACAGCAGCCACATCTA | GGCATTCCTTGTGGCTGTTG | (CAGCAA)5 | 61 | FAM | cds |
| Co261 | ATCGCTTCATGGCATTGTGC | TCAGGATAGGCTTCCTCCGT | (AATGGG)5 | 61 | HEX | utr5 |

Table S3 The environmental variables with variance inflation factors (VIFs) < 10 for 18 populations of *Cephalotaxus oliveri*.

| Pop | Bio2 | Bio8 | Bio9 | Bio10 | Bio12 | Bio13 | EVI | Fpa | LAI | PTC | Slope | Aspect | Altitude | pH | P | K | Na | Mg | Zn | Pb | Fe | Mn |
| --- | --- | --- | --- | --- | --- | --- | --- | --- | --- | --- | --- | --- | --- | --- | --- | --- | --- | --- | --- | --- | --- | --- |
| XF | 7.7 | 22.2 | 4.9 | 24.2 | 1464 | 220 | 0.59 | 0.87 | 4.93 | 33.50 | 8.10 | 158.59 | 697 | 7.88 | 0.35 | 5.33 | 0.86 | 2.03 | 0.04 | 0.02 | 7.41 | 0.37 |
| CX | 8.4 | 26.6 | 5.7 | 26.6 | 1135 | 206 | 0.54 | 0.94 | 5.56 | 43.89 | 14.71 | 95.98 | 231 | 8.14 | 0.37 | 10.77 | 2.34 | 12.72 | 0.06 | 0.04 | 22.40 | 0.61 |
| SX | 7.9 | 22.8 | 2.7 | 22.8 | 1220 | 205 | 0.61 | 0.90 | 5.48 | 44.11 | 13.36 | 83.15 | 340 | 7.45 | 0.47 | 13.08 | 16.74 | 8.30 | 0.09 | 0.02 | 39.03 | 0.74 |
| JS | 8.1 | 22.8 | 4.8 | 24.9 | 1380 | 213 | 0.61 | 0.94 | 6.72 | 50.28 | 24.80 | 150.51 | 291 | 8.04 | 0.81 | 20.54 | 4.73 | 10.80 | 0.08 | 0.03 | 21.76 | 0.74 |
| YM | 7.8 | 21.9 | 3.9 | 24 | 1502 | 241 | 0.62 | 0.92 | 5.61 | 60.17 | 16.50 | 286.90 | 533 | 7.41 | 1.34 | 5.51 | 1.41 | 50.03 | 0.10 | 0.05 | 13.32 | 0.68 |
| HNG | 8.2 | 23.6 | 5.4 | 25.7 | 1438 | 242 | 0.55 | 0.84 | 4.67 | 49.72 | 6.20 | 283.72 | 347 | 7.58 | 1.04 | 4.17 | 0.81 | 62.10 | 0.06 | 0.04 | 8.56 | 0.34 |
| ML | 8 | 23.7 | 8.4 | 24.9 | 1369 | 228 | 0.60 | 0.93 | 6.68 | 46.17 | 2.01 | 204.59 | 635 | 6.92 | 0.43 | 8.49 | 0.97 | 1.94 | 0.08 | 0.03 | 24.61 | 0.48 |
| WYH | 8.6 | 23.9 | 6.5 | 25.8 | 1156 | 198 | 0.54 | 0.96 | 6.08 | 44.72 | 17.85 | 164.87 | 508 | 7.84 | 0.42 | 6.75 | 0.80 | 40.47 | 0.06 | 0.02 | 12.20 | 0.39 |
| FJS | 7.7 | 22 | 4.4 | 23.9 | 1239 | 198 | 0.64 | 0.96 | 6.72 | 50.50 | 33.89 | 239.77 | 739 | 6.16 | 0.34 | 22.72 | 4.58 | 3.79 | 0.11 | 0.02 | 27.87 | 0.41 |
| JFS | 6.6 | 20.3 | 3.9 | 22.3 | 1226 | 182 | 0.57 | 0.95 | 6.69 | 53.67 | 41.39 | 314.55 | 851 | 7.20 | 0.55 | 22.30 | 1.10 | 29.08 | 0.10 | 0.04 | 24.99 | 0.75 |
| LP | 7.2 | 24.6 | 5.9 | 25 | 1283 | 200 | 0.58 | 0.94 | 6.68 | 40.28 | 9.02 | 122.38 | 549 | 8.04 | 0.25 | 13.35 | 1.55 | 3.88 | 0.04 | 0.02 | 11.62 | 0.26 |
| EMS | 7.5 | 22.5 | 6.4 | 23 | 1376 | 336 | 0.60 | 0.93 | 5.58 | 31.50 | 57.90 | 84.37 | 976 | 7.76 | 0.61 | 14.63 | 5.57 | 15.20 | 0.16 | 0.05 | 46.76 | 0.82 |
| FX | 8.8 | 20.3 | 7.5 | 26.4 | 1429 | 230 | 0.62 | 0.95 | 6.70 | 56.44 | 5.77 | 203.88 | 741 | 4.43 | 0.65 | 27.58 | 4.09 | 2.24 | 0.11 | 0.04 | 21.92 | 0.93 |
| WGS | 7.3 | 16.6 | 4.6 | 22.1 | 1860 | 282 | 0.60 | 0.94 | 6.03 | 63.44 | 25.49 | 297.05 | 625 | 4.98 | 0.31 | 26.92 | 1.92 | 3.34 | 0.11 | 0.05 | 22.59 | 0.52 |
| AF | 7.9 | 20.2 | 7.6 | 25.9 | 1646 | 241 | 0.68 | 0.94 | 6.66 | 57.39 | 12.90 | 80.25 | 468 | 5.00 | 0.39 | 28.68 | 4.19 | 2.49 | 0.09 | 0.04 | 17.34 | 0.39 |
| PB | 7.9 | 22.2 | 11.5 | 22.2 | 1477 | 305 | 0.63 | 0.93 | 6.57 | 53.39 | 30.47 | 62.68 | 670 | 5.23 | 0.63 | 19.38 | 1.17 | 1.92 | 0.05 | 0.03 | 31.47 | 0.15 |
| MG | 7.3 | 20.9 | 11 | 20.9 | 1644 | 363 | 0.65 | 0.99 | 6.97 | 61.67 | 20.83 | 267.36 | 1682 | 7.48 | 0.70 | 40.36 | 1.30 | 10.74 | 0.10 | 0.04 | 37.44 | 1.02 |
| HK | 7.9 | 23.3 | 12.8 | 23.3 | 1522 | 316 | 0.64 | 0.91 | 6.47 | 16.67 | 34.31 | 197.96 | 1435 | 5.82 | 1.13 | 8.34 | 0.41 | 8.32 | 0.17 | 0.19 | 43.31 | 1.74 |

Pop, populations; Bio2, mean diurnal temperature range; Bio8, mean temperature of wettest quarter; Bio9, mean temperature of driest quarter; Bio10, mean temperature of warmest quarter; Bio12, annual precipitation; Bio13, precipitation of wettest month; EVI, enhanced vegetation index; FAPAR, fraction of absorbed photosynthetically active radiation; LAI, leaf area index; PTC, percent of tree cover.

Table S4 The result of Hardy-Weinberg test for *Cephalotaxus oliveri* populations

| **Locus** | XF | CX | SX | JS | YM | HNG | ML | WYH | FJS | JFS | LP | EMS | FX | WGS | AF | PB | MG | HK | **Total** |
| --- | --- | --- | --- | --- | --- | --- | --- | --- | --- | --- | --- | --- | --- | --- | --- | --- | --- | --- | --- |
| Co258 | ns | ns | ns | ns | *** | ns | ** | ns | ns | ns | ns | - | ns | ns | ns | *** | - | - | *** |
| Co229 | ns | ns | ns | ns | ns | ns | - | ns | ns | ns | ns | ns | - | ns | ns | ns | - | - | *** |
| Co161 | *** | - | ** | ** | *** | *** | ** | ns | ns | *** | *** | - | - | ns | - | - | - | - | *** |
| Co235 | - | *** | * | ns | ns | ns | ns | - | ns | ns | ns | - | ns | ns | ns | * | - | - | *** |
| Co268 | * | ns | ns | ns | ** | ** | ns | ns | ns | * | ns | ns | ns | ns | *** | *** | ns | ns | *** |
| Co264 | * | ns | ** | ns | ** | * | ns | ns | *** | *** | ** | * | * | ns | ns | ns | ns | ns | *** |
| Co266 | ns | ns | * | ns | *** | *** | ns | ns | ns | * | ns | ns | ns | ** | *** | ns | ns | ** | *** |
| Co111 | ** | ns | ns | * | *** | *** | * | ns | ns | ns | ns | *** | ** | ns | *** | * | * | *** | *** |
| Co146 | ns | ns | ns | - | ns | ns | ns | ns | ns | ns | ns | - | ns | ns | - | ns | ns | ns | ns |
| Co267 | - | - | ns | - | - | ns | - | * | ns | ns | ns | - | - | - | * | - | - | - | *** |
| Co271 | - | ns | *** | ** | * | *** | ns | - | *** | - | *** | - | - | - | ns | - | - | - | *** |
| Co274 | - | ns | *** | *** | ns | ns | *** | - | *** | - | *** | - | - | - | - | *** | - | - | *** |
| Co75 | - | ns | ns | - | ns | - | - | ns | - | - | - | ns | - | - | ns | - | - | - | *** |
| Co20 | - | - | - | - | - | ns | - | ns | - | - | - | - | - | - | - | - | - | - | ** |
| Co14 | - | ns | *** | * | *** | ns | - | ns | ns | *** | ns | ns | ** | ns | *** | - | - | - | *** |
| Co82 | ns | ns | *** | ns | ns | ns | ns | *** | *** | ns | *** | *** | ns | ns | ns | *** | - | - | *** |
| Co43 | ** | ns | *** | - | ns | ns | *** | - | ns | - | ns | - | - | - | - | ns | *** | - | *** |
| Co22 | ** | *** | *** | ns | ns | *** | ** | *** | *** | ns | *** | *** | * | ** | ** | *** | ns | ns | *** |
| Co228 | - | ** | - | - | - | - | - | - | - | - | - | - | - | - | - | ns | - | - | *** |
| Co257 | ns | ns | ns | ns | * | ns | ns | ns | ns | ns | ns | ns | ns | ns | ns | ns | ns | ns | *** |
| Co66 | ns | ns | *** | * | ns | *** | ** | *** | ** | *** | *** | ns | ns | ns | ns | *** | - | - | *** |
| Co224 | ns | - | ns | ns | ns | ns | - | ns | ns | ns | ns | ns | - | ns | ns | ns | - | - | *** |
| Co77 | ns | ns | ns | *** | * | ns | * | ns | ns | *** | *** | ** | ns | * | ** | ns | - | - | *** |
| Co236 | ns | ns | *** | *** | ns | ns | ns | * | *** | ** | ns | ns | * | ** | ns | ns | ns | - | *** |
| Co222 | ns | ns | ns | ns | *** | *** | ns | ns | ns | ns | ns | *** | - | ns | ** | ns | - | - | *** |
| Co244 | ns | * | *** | ** | ns | ** | - | *** | ns | *** | *** | *** | ** | ** | *** | *** | - | - | *** |
| Co234 | ns | ns | ns | ** | ns | ns | ns | ns | * | - | ns | ns | ns | ns | *** | ns | - | - | *** |
| Co261 | ns | ns | ns | * | *** | ns | ns | - | *** | ns | ** | ns | ns | ns | *** | *** | ns | ns | *** |

ns = not significant, * *p* < 0.05, ** *p* < 0.01, *** *p* < 0.001.

Table S5 The pairwise *N*_m_ and *F*_ST_ values between the 18 *Cephalotaxus oliveri* populations.

| pop | XF | CX | SX | JS | YM | HNG | ML | WYH | FJS | JFS | LP | EMS | FX | WGS | AF | PB | MG | HK |
| --- | --- | --- | --- | --- | --- | --- | --- | --- | --- | --- | --- | --- | --- | --- | --- | --- | --- | --- |
| XF | 0.000 | 1.006 | 1.670 | 1.423 | 1.521 | 1.116 | 0.904 | 0.539 | 1.214 | 1.011 | 1.444 | 1.414 | 0.375 | 0.568 | 0.675 | 0.271 | 0.167 | 0.180 |
| CX | 0.199 | - | 3.443 | 1.248 | 1.647 | 1.374 | 0.648 | 0.546 | 1.140 | 1.370 | 2.341 | 1.374 | 0.486 | 0.698 | 0.924 | 0.321 | 0.215 | 0.223 |
| SX | 0.130 | 0.068 | - | 1.681 | 2.444 | 1.814 | 0.955 | 0.730 | 1.460 | 2.213 | 2.503 | 3.291 | 0.779 | 1.160 | 1.599 | 0.416 | 0.318 | 0.340 |
| JS | 0.149 | 0.167 | 0.130 | - | 2.078 | 1.324 | 1.214 | 0.763 | 3.454 | 1.288 | 2.482 | 1.388 | 0.660 | 0.964 | 0.969 | 0.418 | 0.311 | 0.310 |
| YM | 0.141 | 0.132 | 0.093 | 0.107 | - | 6.852 | 1.031 | 1.203 | 2.614 | 2.482 | 3.987 | 2.997 | 0.752 | 1.121 | 1.411 | 0.422 | 0.300 | 0.318 |
| HNG | 0.183 | 0.154 | 0.121 | 0.159 | 0.035 | - | 0.875 | 0.858 | 1.559 | 1.686 | 2.624 | 1.731 | 0.569 | 0.758 | 1.048 | 0.370 | 0.257 | 0.272 |
| ML | 0.217 | 0.278 | 0.207 | 0.171 | 0.195 | 0.222 | - | 0.548 | 1.252 | 0.872 | 1.071 | 0.637 | 0.395 | 0.527 | 0.578 | 0.313 | 0.210 | 0.218 |
| WYH | 0.317 | 0.314 | 0.255 | 0.247 | 0.172 | 0.226 | 0.313 | - | 0.729 | 0.741 | 0.872 | 0.644 | 0.369 | 0.450 | 0.529 | 0.293 | 0.204 | 0.219 |
| FJS | 0.171 | 0.180 | 0.146 | 0.068 | 0.087 | 0.138 | 0.167 | 0.255 | - | 1.446 | 1.937 | 1.072 | 0.646 | 0.793 | 0.871 | 0.371 | 0.268 | 0.290 |
| JFS | 0.198 | 0.154 | 0.102 | 0.163 | 0.092 | 0.129 | 0.223 | 0.252 | 0.147 | - | 2.575 | 1.765 | 0.593 | 0.768 | 0.835 | 0.394 | 0.308 | 0.318 |
| LP | 0.148 | 0.097 | 0.091 | 0.092 | 0.059 | 0.087 | 0.189 | 0.223 | 0.114 | 0.089 | - | 2.154 | 0.530 | 0.786 | 1.317 | 0.416 | 0.297 | 0.307 |
| EMS | 0.150 | 0.154 | 0.071 | 0.153 | 0.077 | 0.126 | 0.282 | 0.280 | 0.189 | 0.124 | 0.104 | - | 0.534 | 0.944 | 1.365 | 0.371 | 0.244 | 0.258 |
| FX | 0.400 | 0.340 | 0.243 | 0.275 | 0.250 | 0.305 | 0.388 | 0.404 | 0.279 | 0.297 | 0.321 | 0.319 | - | 1.115 | 0.728 | 0.263 | 0.163 | 0.168 |
| WGS | 0.306 | 0.264 | 0.177 | 0.206 | 0.182 | 0.248 | 0.322 | 0.357 | 0.240 | 0.246 | 0.241 | 0.209 | 0.183 | - | 0.733 | 0.292 | 0.184 | 0.205 |
| AF | 0.270 | 0.213 | 0.135 | 0.205 | 0.151 | 0.193 | 0.302 | 0.321 | 0.223 | 0.230 | 0.160 | 0.155 | 0.256 | 0.254 | - | 0.357 | 0.249 | 0.253 |
| PB | 0.480 | 0.438 | 0.375 | 0.375 | 0.372 | 0.403 | 0.444 | 0.461 | 0.403 | 0.389 | 0.375 | 0.403 | 0.487 | 0.461 | 0.412 | - | 0.968 | 0.973 |
| MG | 0.599 | 0.538 | 0.440 | 0.446 | 0.455 | 0.493 | 0.543 | 0.551 | 0.482 | 0.448 | 0.457 | 0.506 | 0.606 | 0.576 | 0.501 | 0.205 | - | 1.676 |
| HK | 0.582 | 0.529 | 0.424 | 0.446 | 0.440 | 0.479 | 0.534 | 0.533 | 0.463 | 0.440 | 0.449 | 0.493 | 0.598 | 0.549 | 0.497 | 0.204 | 0.130 | - |

The upper triangle and lower triangle represent the pairwise *N*_m_ and *F*_ST_ values, respectively.

Table S6 Summary results of the Mantel test, partial Mantel test, and multiple matrix regression with randomization analysis (MMRR).

| Test | Parameters | r | β | *p* |
| --- | --- | --- | --- | --- |
| Mantel | IBD：Gen vs. Geo | 0.670 |  | 0.001^**^ |
|  | IBE：Gen vs. Env | 0.391 |  | 0.024^*^ |
|  | Geo vs. Env | 0.3988 |  | 0.001^**^ |
| Partial Mantel | Gen vs. Geo \| Env | 0.609 |  | 0.001^**^ |
|  | Gen vs. Env \| Geo | 0.183 |  | 0.142^ns^ |
| MMRR | Gen vs. Geo |  | 0.0007 | 0.001^**^ |
|  | Gen vs. Env |  | 0.0743 | 0.017^*^ |
|  | Gen vs. Geo + Env |  | Geo: 0.0007 | 0.001^**^ |
|  |  |  | Env: 0.0276 | 0.215^ns^ |

Gen, genetic distance (*F*_ST_); Geo, geographic distance; Env, environmental distance; partial-Mantel tests: X ~ Y(Z) is the correlation between X and Y matrices, controlling for Z; β, the effects of geographic/environmental distance on genetic distance; significance: * *p* < 0.05, ** *p* <0.01, ns = not significant.

Table S7 The significant environmental and geographic variables retained by the initial step-forward selection method.

| Variable | Adjusted *R*^2^ | AIC | F | *p*-value |
| --- | --- | --- | --- | --- |
| *Environmental variables* | | | | |
| bio9 | 0.09869 | -392.18 | 37.0247 | 0.001 |
| bio10 | 0.13071 | -403.12 | 13.0799 | 0.001 |
| pH | 0.15542 | -411.65 | 10.5689 | 0.001 |
| Mg | 0.17555 | -418.62 | 8.9588 | 0.001 |
| bio13 | 0.19659 | -426.17 | 9.5108 | 0.001 |
| Aspect | 0.21347 | -432.2 | 7.9547 | 0.001 |
| bio2 | 0.23057 | -438.48 | 8.1791 | 0.001 |
| FAPAR | 0.24402 | -443.32 | 6.7269 | 0.001 |
| EVI | 0.25628 | -447.75 | 6.2908 | 0.001 |
| Na | 0.26887 | -452.42 | 6.5139 | 0.001 |
| LAI | 0.28078 | -456.87 | 6.2797 | 0.001 |
| bio8 | 0.29122 | -460.73 | 5.6833 | 0.001 |
| K | 0.30247 | -465.06 | 6.1164 | 0.001 |
| P | 0.3149 | -470.04 | 6.7319 | 0.001 |
| Pb | 0.32263 | -472.83 | 4.5957 | 0.001 |
| Fe | 0.33294 | -476.95 | 5.8524 | 0.001 |
| *Geographic variables* | | | | |
| PCNM1 | 0.10596 | -394.85 | 39.9908 | 0.001 |
| PCNM2 | 0.14318 | -407.89 | 15.2501 | 0.001 |
| PCNM3 | 0.16538 | -415.56 | 9.6981 | 0.001 |
| PCNM7 | 0.18471 | -422.31 | 8.7301 | 0.001 |
| PCNM4 | 0.19922 | -427.25 | 6.8866 | 0.001 |
| PCNM5 | 0.21227 | -431.7 | 6.3696 | 0.001 |
| PCNM9 | 0.22278 | -435.15 | 5.3684 | 0.001 |
| PCNM6 | 0.2323 | -438.24 | 4.9894 | 0.001 |
| PCNM10 | 0.24019 | -440.68 | 4.3351 | 0.001 |

P, soil P content; Fe, soil Fe content; Mg, soil Mg content; K, soil K content; Pb, soil Pb content; Na, soil Na content; pH, soil pH; Bio9, mean temperature of driest quarter; Bio10, mean temperature of warmest quarter; Bio8, mean temperature of wettest quarter; Bio12, annual precipitation; Bio13, precipitation of wettest month; Bio2, mean diurnal temperature range; LAI, leaf area index; FAPAR, fraction of absorbed photosynthetically active radiation; EVI, enhanced vegetation index.

Table S8 Outlier loci associated with environmental variables identified by the linear mixed-effects model (LMM)

| locus_allele | bio2 | bio8 | bio9 | bio12 | bio13 | Fpa | LAI | PTC | Aspect | Altitude | pH | P | K | Na | Mg | Zn | Pb | Fe | Mn | Total |
| --- | --- | --- | --- | --- | --- | --- | --- | --- | --- | --- | --- | --- | --- | --- | --- | --- | --- | --- | --- | --- |
| Co161_177 |  |  |  |  | * |  |  |  | * |  |  |  |  |  |  |  |  |  |  | 2 |
| Co161_178 |  |  |  |  | * |  |  |  | * |  |  |  |  |  |  |  |  | * |  | 3 |
| Co235_249 |  | * |  |  |  |  |  |  |  |  |  |  |  |  |  |  |  |  |  | 1 |
| Co235_258 |  | * |  |  |  |  |  |  |  |  |  |  |  |  |  |  |  |  |  | 1 |
| Co235_256 |  |  |  |  |  |  |  |  |  | *** |  |  |  |  |  | * | * |  | *** | 4 |
| Co235_247 |  |  | * |  |  |  |  |  |  |  |  |  |  |  |  |  |  |  | * | 2 |
| Co235_244 |  |  |  |  |  |  |  |  |  | * |  |  |  |  |  |  |  |  | *** | 2 |
| Co274_251 |  |  | * |  |  |  |  |  |  |  |  |  |  |  |  |  |  |  |  | 1 |
| Co274_243 |  |  |  |  |  |  |  |  |  | * |  |  |  |  |  |  |  |  | * | 2 |
| Co274_240 |  |  |  |  |  |  |  |  |  |  |  |  |  | *** |  |  |  |  |  | 1 |
| Co274_244 |  |  |  |  |  |  |  |  |  | * |  |  |  |  |  |  |  |  |  | 1 |
| Co14_267 |  | ** | * | ** | ** |  |  |  |  |  | *** | ** | * |  |  |  |  |  |  | 6 |
| Co14_261 |  |  | ** | * | ** |  |  |  |  | * |  |  |  | * |  |  |  |  |  | 5 |
| Co14_255 |  |  |  |  | * |  |  |  |  |  |  |  |  |  |  |  |  |  |  | 1 |
| Co14_279 |  | *** |  | * |  |  |  |  |  |  | ** |  |  |  |  |  |  |  |  | 3 |
| Co14_273 |  | * |  |  |  |  |  |  |  |  | ** |  |  |  |  |  |  |  |  | 2 |
| Co14_256 |  |  |  |  |  |  |  |  |  |  |  | ** |  |  |  |  |  |  |  | 1 |
| Co14_262 |  |  |  |  |  |  |  |  |  |  |  | ** |  |  | * |  |  |  |  | 2 |
| Co14_266 | ** |  |  |  |  |  |  |  | * |  |  |  |  |  |  |  |  |  |  | 2 |
| Co14_272 |  | *** |  | *** |  |  |  | * |  |  | *** |  | * |  |  |  |  |  |  | 5 |
| Co14_278 | * |  |  |  |  |  |  |  |  |  | * |  |  |  |  |  |  |  |  | 2 |
| Co224_223 |  |  |  |  |  |  |  |  |  |  |  |  |  |  |  |  |  |  | * | 1 |
| Co224_235 |  |  |  |  |  |  |  |  |  |  |  | *** |  |  | *** |  |  |  |  | 2 |
| Co224_218 |  |  |  |  |  |  |  |  |  |  |  | ** |  |  | * |  |  |  |  | 2 |
| Co222_217 |  | ** | ** | ** | * |  |  |  |  |  | *** |  | * |  |  |  |  |  |  | 6 |
| Co222_223 |  | ** |  | * |  |  |  |  |  |  | *** |  |  |  |  |  |  |  |  | 3 |
| Co222_224 |  |  |  |  |  |  |  |  | * |  |  |  |  | ** |  |  |  |  |  | 2 |
| Co222_211 |  |  |  |  |  |  |  |  |  | ** |  |  |  |  |  | * | * |  | *** | 4 |
| Co222_230 |  |  |  |  |  | ** | ** |  |  |  |  | * |  |  | *** |  |  |  |  | 4 |
| Co222_229 |  |  |  |  |  | ** | * |  |  |  |  |  |  |  | ** |  |  |  |  | 3 |
| Co222_199 |  |  |  |  |  |  |  |  |  | * |  |  |  |  |  |  |  |  | *** | 2 |
| Total | 2 | 8 | 5 | 6 | 6 | 2 | 2 | 1 | 4 | 7 | 7 | 5 | 3 | 3 | 5 | 2 | 2 | 1 | 7 |  |

*, ** and *** represent p<0.05, p<0.01 and p<0.001, respectively.
